# Fellowship exit examination in orthopaedic surgery in the commonwealth countries of Australia, UK, South Africa and Canada. Are they comparable and equivalent? A perspective on the requirements for medical migration

**DOI:** 10.1080/10872981.2018.1537429

**Published:** 2018-10-29

**Authors:** Erik Hohmann, Kevin Tetsworth

**Affiliations:** a Department of Orthopedic Surgery and Sports Medicine, Valiant Clinic/Houston Methodist Group, Dubai, United Arab Emirates; b Faculty of Health Sciences, Medical School, University of Pretoria, Pretoria, South Africa; c Department of Orthopaedic Surgery, Royal Brisbane Hospital, Herston, Australia; d Department of Surgery, School of Medicine, University of Queensland, Brisbane, Australia

**Keywords:** Orthopaedic surgery training, syllabus, surgical curriculum, compatible training and fellowship examination, medical migration

## Abstract

International migration of healthcare professionals has increased substantially in recent decades. In order to practice medicine in the recipient country, International Medical Graduates (IMG) are required to fulfil the requirements of their new countries medical registration authorities. The purpose of this project was to compare the final fellowship exit examination in Orthopaedic Surgery for the UK, Australia, Canada and South Africa.

The curriculum of the Australian Orthopaedic Association (SET) was selected as a baseline reference. The competencies and technical modules specified in the training syllabus, as well as the specifics of the final fellowship examination as outlined in SET, were then compared between countries.

Of the nine competencies outlined in SET, the curricula of the UK, South Africa and Canada were all compatible with the Australian syllabus, and covered 97.7%, 86% and 93%, respectively, of all competencies and sub-items. The final fellowship examinations of Australia, South Africa and the UK were all highly similar in format and content. The examination in Canada was substantially different, and had two written sessions but combined the oral and clinical component into a structured OSCE using standardized patients and the component included unmanned stations. There were no significant differences for completion certificate of training and/or board certification observed between these countries.

The results of this study strongly suggest that core and technical competencies outlined in the training and education curriculum and the final fellowship examination in Orthopaedic Surgery in Australia, South Africa and the UK are compatible. Between country reciprocal recognition of these fellowship examinations should not only be considered by the relevant Colleges, but should also be regulated by the individual countries health practitioner registration boards and governing bodies.

## Introduction

International migration of healthcare professionals was initially recognized in the 1940s when medical doctors, mainly from Europe, emigrated to the UK (UK) and the USA (US) [1,2]. Common reasons for this migration movement included low wages, limited career opportunities and additional economic factors such as job security and professional development opportunities [1,3,4]. Mullan reported that 23–28% of the workforce in the USA, the UK, Canada and Australia constitute international medical graduates (IMG) [5]. Furthermore, there is a major exchange of medical doctors between these developed countries, and British doctors form the largest contingent of IMGs in both Canada and Australia. South African doctors form the 4^th^ largest contingent in the UK and Australia, and the 2^nd^ largest in Canada [1,5].

In order to practice medicine in the recipient country, IMGs are required to fulfil the requirements of that countries medical registration authorities. For example, for registration in the UK by the General Medical Council (GMC), IMGs from non-European Union member countries have to complete the PLAB (Professional and Linguistic Assessment Board) [6]. In Australia, different pathways for general registration have been developed by the Australian Health Practitioner Regulation Agency (AHPRA) [7]. While maintenance of standards are important, increasing globalization has led many medical schools, across many countries, to adopt common standards for clinical teaching, professionalism and assessment [8,9]. Registration requirements for IMGs from these medical schools may, therefore, not be necessary.

For specialist consultants, registration requirements differ from the common registration pathway, and they must become Fellows of the relevant specialist colleges. This generally includes a period of supervision, and may require they sit the final fellowship examination in their specialty [6,10]. Syed has reported the duration of orthopaedic training across the UK, Canada, the USA and Australia are very similar, and concluded orthopaedic training in the UK remains comparable to these countries [11]. If the final fellowship exit examinations in these countries are also similar, reciprocal recognition by the Colleges and Registration Agencies would not only be more cost-effective but would also reduce the bureaucratic burden for both the migrating specialist and recipient country.

The purpose of this project was, therefore, to compare the final fellowship examination in Orthopaedic Surgery for the Commonwealth Countries of the UK, Australia, Canada and South Africa. We hypothesized that there would be no significance between country differences, making these examinations comparable.

## Methods

The websites of the Royal Australasian College of Surgeons [12,13], Australian Orthopaedic Association [14,15], Intercollegiate Joint Committee on Intercollegiate Examinations of the UK [16], British Orthopaedic Association [17], Royal College of Physicians and Surgeons of Canada [18–21] and the Colleges of Medicine of South Africa [22] were accessed and searched using the following terms: orthopaedic training syllabus, specialist training in orthopaedic surgery, orthopaedic training, objectives of training in orthopaedic surgery, orthopaedic surgical education and curriculum, regulations for education and training in orthopaedic surgery, examination in orthopaedic surgery, fellowship examination in orthopaedic surgery, college orthopaedic surgery examination. If detailed information was not displayed on their websites, the respective Colleges were contacted directly via email.

All available documents were screened to establish entry criteria into higher orthopaedic surgical training (as an accredited training registrar), and the length and curriculum of required training. These criteria served to establish comparability of the surgical training, and to establish whether there were any significant discrepancies in the training process that could result in the exit examination assessing a different syllabus. This comparison was performed to exclude the possibility that, despite a similarly structured and comparable exit assessment, knowledge of a different syllabus was being tested during the final fellowship examination. For the purpose of comparison of the syllabus and surgical training between the four different countries, the ‘Surgical Education and Training – Orthopaedic Surgery Competency Based Curriculum’ of the Australian Orthopaedic Association was selected as a baseline reference [14].

The Australian curriculum was selected as the baseline based because this syllabus provides a clear and concise list of the competencies that must be achieved during the training period, and provides the most explicit description of the technical modules covered during training. This facilitated a structured search of the other countries’ curricula, with comparison against these criteria. The competencies listed in the Australian syllabus were: medical expertise, technical expertise, judgement-decision making, communication, collaboration, management and leadership, health advocacy, scholar and teacher, and professionalism. These competencies were further subdivided into subcategories; for example ‘medical expertise’ was defined as ‘access & apply relevant knowledge to clinical practice’ and the following criteria were assessed to define competency in this category: maintain currency of knowledge, apply scientific knowledge in practice, recognize and solve real life problems. The technical modules were divided into a common core syllabus of surgery, orthopaedic principles and basic science, paediatrics, spine, shoulder and elbow, hand, hip, knee, arthroplasty, trauma, foot and ankle, tumour and genetic/metabolic/neurological disorders. For example, the arthroplasty module listed clinical competencies for total and unicompartmental knee arthroplasty, total hip arthroplasty, revision arthroplasties, upper limb, and foot and ankle arthroplasties using the following sub-items: knowledge (anatomy, pathophysiology, biomechanics, clinical presentation, current controversies), and skills (examination, investigation, treatment, attitudes and additional competencies) [14].

The curricula and syllabuses for the orthopaedic surgery training program in the UK, South Africa and Canada were then also reviewed for all competencies and requisite technical modules. When reviewing the curricula of the other three Commonwealth Countries comparability was not based on finding the identified keywords for the core and technical competencies but to instead identify whether these key-terms were addressed and described in those publications. Comparability was defined if at least 80% of the competencies and technical modules, and their subcategories, for each of these three countries matched those identified in the Australian curriculum. The websites were then searched for details regarding the final fellowship examination in Orthopaedic Surgery for each of these countries and again compared to Australia as the standard, with at least 80% agreement defined as comparable.

Similar to assessment of the curricula and syllabus, and to be with consistent with the methodology, the Australian Final Fellowship Examination in Orthopaedic Surgery was used to investigate comparability between countries. Comparability criteria here were defined more strictly, considering the exit examination is regarded as the final hurdle to allow an orthopaedic surgeon to practice independently. While this criterion does not specifically assess the quality of training or examination standards between individual surgeons, it does serve as a reliable tool to assess substantial comparability between international medical graduates as suggested by the policy of the Royal Australasian College of Surgeons [12].

The final fellowship exit examination in Orthopaedic Surgery in Australia is currently comprised of multiple components, beginning with a two hour written multiple-choice test. This is followed by a two hour written exam including both essays and multiple short questions. There are then three viva voce 30-min oral examinations, including one on diagnostic investigations, and a further two operative surgery sessions. Finally, the clinical component consists of two 30-min sessions including up to five clinical cases per session. To be comparable to this Australian standard, the final fellowship examination in the other three countries needed to satisfy the minimum criteria of at least two written components of at least two hours duration, including either multiple choice questions, short essay questions, essays or any combination of these. For the oral component, a minimum of three viva sessions with a minimum of 30-min duration were defined as comparable. Finally, for the clinical component a minimum of three clinical examination sessions of at least 30-min duration were required to accept comparability. For the written and oral component of the fellowship examination to be considered comparable the entire syllabus had to be covered during the examination sessions.

## Results

Entry criteria for higher surgical training varied between countries (Table 1). However, the requirement to pass an entry examination was similar for all four countries.

**Table 1.** Entry criteria into higher surgical orthopaedic training.

UK	South Africa	Canada	Australia
FRCS (Tr&Orth)	FCS (Orth)	FRCSC	FRACS
Intercollegiate MRCS	Primary Fellowship Examination (Basic Sciences)	Completion of Surgical Foundation Examination (Basic Sciences)	Surgical Science Examination
	Intermediate Fellowship Examination (Principles of Surgery)		
2 foundation years		24 months of approved foundation training	
	3 Months Term in ICU	Minimum of 4 Weeks ICU Term	Care of the Critically Ill Course
	3 Months Term in General Surgery	Minimum 4 Weeks of Trauma Management Term	At least 6 months of Orthopaedic Experience
			2 months emergency term
Workplace based assessments			4 Quarterly Satisfactory Assessments
	Basic Surgical Skills Course		Orthopaedic Principles and Basic Science Module
			EMST/ACLS
			Critical Literature and Research Course (desirable)

Nine competencies were described in the ‘Surgical Education and Training – Orthopaedic Surgery Competency Based Curriculum’ of the Australian Orthopaedic Association. The curricula of the UK, South Africa and Canada were directly comparable to the Australian syllabus, and mentioned 97.7%, 86% and 93%, respectively, of all competencies and sub-items (Table 2).

**Table 2.** Core competencies.

		UK	South Africa	Canada	Australia
	Subitems	FRCS (Tr&Orth)	FCS (Orth)	FRCSC	FRACS
Medical Expertise	3	3 (100%)	2 (66%)	3 (100%)	3 (100%
Technical Expertise	7	7 (100%)	6 (86%)	6 (86%)	7 (100%)
Judgement/Decision Making	9	8 (89%)	8 (89%)	8 (89%)	9 (100%)
Communication	4	4 (100%)	3 (75%)	4 (100%)	4 (100%)
Collaboration	4	4 (100%)	4 (100%)	4 (100%)	4 (100%)
Management and Leadership	3	3 (100%)	3 (100%)	3 (100%)	3 (100%)
Health Advocacy	2	2 (100%)	2 (100%)	2 (100%)	2 (100%)
Scholar and Teacher	4	4 (100%)	4 (100%)	4 (100%)	4 (100%)
Professionalism	7	7 (100%)	5 (71%)	6 (86%)	7 (100%)
Total	43	42 (97.7%)	37 (86%)	40 (93%)	43 (100%)

Thirteen technical modules were described in the ‘Surgical Education and Training – Orthopaedic Surgery Competency Based Curriculum’ of the Australian Orthopaedic Association. The curricula of the UK and South Africa were both highly comparable, and mentioned all 13 modules; Canada mentioned 12 of the 13 modules, but did not mention the common core syllabus of surgery (Table 3).

**Table 3.** Technical modules.

	UK	South Africa	Canada	Australia
Technical Modules	FRCS (Tr&Orth)	FCS (Orth)	FRCSC	FRACS
Common Core Syllabus of Surgery	yes	yes	not mentioned	yes
Orthopaedic Principles and Basic Science	yes	yes	yes	yes
Paediatric	yes	yes	yes	yes
Spine	yes	yes	yes	yes
Shoulder and Elbow	yes	yes	yes	yes
Hand	yes	yes	yes	yes
Hip	yes	yes	yes	yes
Knee	yes	yes	yes	yes
Arthroplasty	yes	yes	yes	yes
Trauma	yes	yes	yes	yes
Foot and Ankle	yes	yes	yes	yes
Tumour	yes	yes	yes	yes
Genetic/Metabolic/Neurological	yes	yes	yes	yes

The duration of training was comparable for all four countries (Table 4). The longest duration training is required in the UK where a minimum of eight years, including internship/foundation years, was necessary for certification. In Canada the minimum duration of training was six years; in both South Africa and Australia the minimum training duration was 6.5 years (Table 4).

**Table 4.** Duration of training.

UK	South Africa	Canada	Australia
FRCS (Tr&Orth)	FCS (Orth)	FRCSC	FRACS
2 Foundation Years – Minimum of 10 Months Rotation in Orthopaedic Surgery	1 Year Internship	1 Year Residency/Intern	1 Year Internship
	12 Months of Orthopaedic Experience		6 Months Orthopaedic Experience
	3 Months General Surgery Rotation		
	3 Months ICU Rotation		
6 Years of Orthopaedic Registrar Training	4 Years of Orthopaedic Registrar Training	5 years of Orthopaedic Residency Training	5 Years of Orthopaedic Registrar Training

The final fellowship examinations of Australia, South Africa and the UK were very similar in structure. In comparison to Australia, South Africa required candidates to complete three rather than two written sessions; the UK required candidates to complete four rather than three oral sessions, exceeding the defined criteria for comparability. Canada combined the oral and clinical component into a structured OSCE (objective structured clinical examination), with 11 stations of 15 min each. The Canadian College used standardized patients and the component included unmanned stations. This approach was significantly different to the other three countries, and was deemed not comparable with the defined criteria (Table 5).

**Table 5.** Final fellowship examination in orthopaedic surgery.

	UK	South Africa	Canada	Australia
Component	FRCS (Tr&Orth)	FCS (Orth)	FRCSC	FRACS
Written 1	Multiple Choice (2 h)	3 essays (3 h)	Multiple Choice (115 questions)	Multiple Choice (2 h)
Written 2	Multiple Choice (2,5 h)	3 essays (3 h)	Short Answer Questions (40–60)	Essays and Short Answers (2 h)
Written 3		3 essays (3 h)		
Oral 1	Applied Basic Sciences (30 min)	Orthopaedic Pathology (30 min)	OSCE (3 h)	Investigations (30 min)
Oral 2	Adult Orthopaedics and Spine (30 min)	Reconstructive Orthopaedics (30 min)	Eleven Stations (15 min each)	Operative Surgery (30 min)
Oral 3	Trauma including Spine (30 min)	Orthopaedic Trauma (30 min)	Manned: Standardized Patient or Oral	Operative Surgery (30 min)
Oral 4	Paediatrics and Hands (30 min)		Unmanned: Written Answers	
Clinical 1	Intermediate Cases (2x15 min)	Long Case (30 min)		5 Short Cases (30 min)
Clinical 2	Short Cases (2x15 min Upper Limb 2 × 15 min Lower Limbs 2 × 15 min	Short Cases (30 min)		5 Short Cases (30 min)

The requirements for completion certificate of training and board certification are outlined in Table 6. There were no significant differences observed between these four countries.

**Table 6.** Board certification requirements.

UK	South Africa	Canada	Australia
FRCS (Tr&Orth)	FCS (Orth)	FRCSC	FRACS
Completion of FRCS (Tr&Orth)	Completion of FCS (Orth)	Completion of FRCSC	Completion of FRACS
2 Foundation Years	1 Year Internship	1 Year Internship	1 Year Internship
	18 Months Medical Officer		6 Months Orthopaedic Experience
6 Years Orthopaedic Registrar	4 Years Orthopaedic Registrar	5 Years Orthopaedic Registrar	5 Years Orthopaedic Registrar
Complete Surgical Logbook			
Sufficient and Satisfactory Workplace Based Assessments			Quarterly Satisfactory Assessments
Evidence of Involvement in Quality Improvement			
Evidence of Publications and Presentations	Completion of Masters Project in Orthopaedic Surgery (MMed)	Completion of Research Project	Successful Completion and Submission of a Research Project
ATLS			

## Discussion

The most important finding of this study was that the entry criteria into higher surgical training, technical modules and competencies (as outlined in the SET Curriculum of the Australian Orthopaedic Association), the duration of training, and board certification for all four of these commonwealth countries are comparable. Most importantly, the final fellowship exit examination in the field of Orthopaedic Surgery for South Africa, Australia and the UK are clearly comparable to one other.

Migration of medical professionals is part of today’s social, economic and professional globalization trend [1–5,23]. Medical migration from low and middle-income countries is a large scale and long-standing phenomenon that is detrimental to the health systems in the donor countries [23]. Canada, the USA and the UK have been the main beneficiaries over the past half-century [5]. Recruitment from low-income and developing countries remains ethically questionable, and the WHO has developed a global code of practice on the international recruitment of health personnel [24]. However, migration between developed countries is also common, and British and South African doctors form a large contingent of doctors in both Canada and Australia [1,5].

Registration with professional bodies serves several important functions, and helps to assure the registered individuals are fit to practice medicine [25]. Maintaining a contemporaneous registry of qualified doctors facilitates simultaneous control of entry to the register of recognized physicians. Regulatory bodies can also foster good medical practice and the principles and values that underpin this concept. Furthermore, they can help establish and promote high standards of medical education and training within the profession. Finally, registration bodies should be best prepared to deal firmly and fairly with those doctors whose fitness to practise is in doubt [26]. Vries et al. compared 10 medical regulatory systems and concluded that these systems were all surprisingly similar, with the exception of the requirement of revalidation [27]. These findings would support reciprocal registration within these countries without the need to sit any registration examinations, unless an individual has been removed from the register previously, or there are insufficient details or documents to support that individuals’ registration.

Registration as a specialist generally requires a local fellowship within the specialty, and it is uncommon to accept specialist qualification from other countries [6,10,12,19]. In order to practice independently, overseas trained doctors are often required to sit the local fellowship examination [6,10,12,28]. The fellowship examinations are governed and executed by the local specialist colleges. These colleges are professional bodies are typically self-governed, and their policies are not regulated by any federal legislation [29]. Impartiality, procedural fairness, and natural justice may thus not be guaranteed without government oversight. The pass rate for overseas trained specialists presenting for these examinations is significantly lower when compared to the locally trained specialists [30,31]. Rogers et al. suggested the lower pass rates in Emergency Medicine for overseas candidates are directly related to exposure to members of the court of examiners, and these are mainly located in the larger tertiary centres [32]. Raddatz et al. reported that non-fellowship trained examinees score systematically lower than their locally-qualified counterparts, suggesting the reasons behind the differences are not related to the stability of the examination construct but lies somewhere else [33]. The Australian Orthopaedic Association acknowledges that the ability of IMG’s to pass the exam is more difficult, particularly in regional areas where access to teaching may be restricted [34]. A recent survey has suggested that overseas trained psychiatrists in Australia believe they were filling positions where locally qualified fellows do not wish to work, and considered the required examination process flawed and inaccurate [1,35].

The need for an exit examination to provide a common denominator for trainees, and to confirm a high standard of surgical practice, is obvious [36]. However, the need to repeat a final fellowship examination in a particular speciality specifically to regulate registration with the medical board and thereby control access to independent practice can be questioned. Formal assessments are not a panacea, and important elements of competence such as teamwork, ethical behaviour, communication, clinical judgement and surgical skills are not assessed under examination conditions, and do not reflect behaviour at work [37]. Moreover determination of a valid ‘pass mark’ remains an elusive goal [37]. Hays & Morgan compared general practice training models in 12 countries and noticed strong similarities between training in Australia and training in many other countries, concluding that reciprocal recognition should be allowed with at least some of these countries [38].

Other modalities may be more useful if the governing and registration authorities see the need for a mechanism to evaluate an overseas trained applicant. Nachbauer reported on the value of an exit assessment in vascular surgery, and reported a prerequisite for assessment should be the completion of training in the home country [36]. Two to three eminent vascular surgeons from each country review the documentation of each candidate; and candidates would only fail by general agreement. Nachbur suggested that this robust process avoids favouritism, and is already commonly employed in Belgium, Greece, Italy, Spain and Switzerland [36]. Bhatti and Cummings suggested various instruments such as a 360-degree evaluation, of outcomes, and performance-based evaluations of surgical practice [39]. These suggestions may certainly be more reliable than the formal examination process currently employed to assess postgraduate trainees concluding their formative years [1]. The College Royal Australian and the New Zealand College of Psychiatrists has recently introduced workplace-based assessments as a pathway to fellowship [35,40]. However, this option is only available to those trained in Anglo-Saxon countries, and may reinforce the perception of this policy as discriminatory and divisive [35,40].

The question remains whether these measures are even necessary. The results of this project suggest that they are obsolete for those overseas trained specialists with qualifications from countries with comparable final examinations. Within the specialty of Orthopaedic Surgery, this includes the Commonwealth countries of South Africa, Australia and the UK.

Ultimately, political action will be instrumental to drive any such change. In Australia, a recent parliamentary enquiry into registration and support for overseas trained doctors has been concluded, and 45 recommendations were made in the final report [41]. These recommendations covered a broad range, but begin with publishing agreed upon definitions of levels of comparability. They further suggest developing objective guidelines on the Colleges’ websites as to how overseas qualifications, skills, and experience are taken into account, as well as establishing an overarching independent appeal mechanism to review decisions of a specialist medical college. However, it appears these recommendations have not been acted upon yet. The latest policy by the Royal Australasian College of Surgeons from June 2015 still does not list definitions of levels of comparability or objective guidelines, and fails to specify how overseas qualifications, skills and experience are taken into consideration [12].

This study has certain limitations. The accuracy of this study relies on the information gained via access to the colleges, other professional bodies and organizations. Orthopaedic surgery fellowship examination and training details may have changed or may not have been published on these websites. This project cannot claim to be a complete qualitative analysis of the training and examination processes involved. However, it can be safely assumed that the provided information on the websites was reliable and valid at the time of access. We, therefore, believe that these comparisons are both reliable and accurate. In contrast to the UK, South Africa and Canada, the Australian training and education curriculum provided a list of competencies which allowed a check of the other curriculi against these specified criteria. As the curricula of other three countries are written in a more narrative fashion, it required intense study to compare these competencies against the Australian curriculum. It is acknowledged that certain criteria may have been missed during the screening which may introduce an element of bias.

## Conclusions

The results of this study strongly suggest that core cognitive and technical competencies outlined in the training/education curriculum and subjected to scrutiny during the final fellowship examination in Orthopaedic Surgery in Australia, South Africa and the UK are compatible. Between country reciprocal recognition of these fellowship examinations should not only be considered by the relevant Colleges, but should also be regulated by the individual countries health practitioner registration boards and governing bodies.
